# Leaky-gut enhanced lupus progression in the Fc gamma receptor-IIb deficient and pristane-induced mouse models of lupus

**DOI:** 10.1038/s41598-019-57275-0

**Published:** 2020-01-21

**Authors:** Arthid Thim-uam, Saowapha Surawut, Jiraphorn Issara-Amphorn, Thiranut Jaroonwitchawan, Pratsanee Hiengrach, Piraya Chatthanathon, Alisa Wilantho, Naraporn Somboonna, Tanapat Palaga, Prapaporn Pisitkun, Asada Leelahavanichkul

**Affiliations:** 1 Interdisciplinary Program of Biomedical Sciences, Graduate School, Chulalongkorn University, Bangkok, Thailand; 2 Department of Biology, Faculty of Science and Technology, Rambhai Barni Rajabhat University, Chanthaburi Province, 22000 Thailand; 3 Department of Microbiology, Faculty of Medicine, Chulalongkorn University, Bangkok, Thailand; 4 Department of Microbiology, Faculty of Science, Chulalongkorn University, Bangkok, Thailand; 5 Microbiome Research Unit for Probiotics in Food and Cosmetics, Chulalongkorn University, Bangkok, Thailand; 6 Genome Technology Research Unit, National Center for Genetic Engineering and Biotechnology, Khlong Luang, Pathum Thani 12120 Thailand; 7 Division of Allergy, Immunology, and Rheumatology, Department of Medicine, Faculty of Medicine, Ramathibodi Hospital, Mahidol University, Bangkok, Thailand; 8 Translational Research In Inflammation and Immunology Research Unit (TRIRU), Department of Microbiology, Chulalongkorn University, Bangkok, Thailand

**Keywords:** Autoimmune diseases, Medical research

## Abstract

The influence of gut-leakage or gut-microbiota upon lupus progression was explored in 2 lupus mouse models. Pristane, administered in 4-wk-old wild-type (WT) female mice, induced lupus characteristics at 24-wk-old similar to the lupus-onset in FcGRIIb−/− mice. Gut-microbiota alteration was induced by co-housing together with the gavage of feces from 40-wk-old FcGRIIb−/− mice (symptomatic lupus). On the other hand,  gut-leakage was induced  by dextran sulfate solution (DSS). DSS and gut-microbiota alteration induced high serum anti-dsDNA immunoglobulin (Ig) as early as 30 days post-DSS only in FcGRIIb−/− mice. DSS, but not gut-microbiota alteration, enhanced lupus characteristics (serum creatinine and proteinuria) in both lupus models (but not in WT) at 60 days post-DSS. Indeed, DSS induced the translocation of molecular components of gut-pathogens as determined by bacterial burdens in mesenteric lymph node (MLN), endotoxemia (gut-bacterial molecule) and serum (1→3)-β-D-glucan (BG) (gut-fungal molecule) as early as 15 days post-DSS together with enhanced MLN apoptosis in both WT and lupus mice. However, DSS induced spleen apoptosis in FcGRIIb−/− and WT mice at 30 and 60 days post-DSS, respectively, suggesting the higher impact of gut-leakage against spleen of lupus mice. In addition, macrophages preconditioning with LPS plus BG were susceptible to starvation-induced apoptosis, predominantly in FcGRIIb−/− cell, implying the influence of gut-leakage upon cell stress. In summary, gut-leakage induced gut-translocation of organismal-molecules then enhanced the susceptibility of stress-induced apoptosis, predominantly in lupus. Subsequently, the higher burdens of apoptosis in lupus mice increased anti-dsDNA Ig and worsen lupus severity through immune complex deposition. Hence, therapeutic strategies addressing gut-leakage in lupus are interesting.

## Introduction

Systemic lupus erythematosus (SLE) is a common autoimmune disease with multi-organ involvement^1^. Fc gamma receptor IIb (FcGRIIb) dysfunction polymorphism associates with SLE, particularly in Asian populations^2^, possibly due to malaria-based selection pressure^3^. Indeed, the overexpression of FcGRIIb, either in autoimmune-prone mouse strains or wild-type (WT) animals, heightened the threshold for induction of autoimmune disease^4^. The defects of FcGRIIb, the only inhibitory receptor in FcGR family, induce exaggerated immune responses and cause lupus^5^. As such, FcGRIIb−/− mouse is an established lupus mouse model with lupus characteristics as early as 20–24 wks old and develops full-blown lupus after 32–40 wks old^5,6^. In parallel, a single peritoneal injection of pristane (2, 6, 10, 14-tetramethypentadecane), a hydrocarbon substance derived from shark liver-oil, causes chronic peritoneal inflammation and induces lupus characteristics as early as 20–24 wks after the injection^7^. The age-related lupus manifestations in either FcGRIIb−/− or pristane mice allow the exploration of lupus in asymptomatic and symptomatic status.

Although the association between gut-microbiota composition and disease progression of lupus has been demonstrated^8^, the information of gut-leakage against lupus is still limited. In a normal situation, gut-barrier is a natural protection that safeguards the translocation of pathogen associated molecular patterns (PAMPs) and viable organisms from gut into blood circulation^9^. The defect of gut-permeability causes the translocation of PAMPs and gut-organisms that induces systemic immune responses. In parallel, some specific gut-bacterial microbiota have been reported to induce lupus, especially with the translocation gut-pathobiont (pathogenic bacteria)^10^. Indeed, spontaneous gut-leakage in active lupus in mice and patients due to the deposition of circulating immune complexes (CIC) in gut are reported^11–13^. Because i) GI tract is the endogenous source of endotoxin and (1→3)-β-D-glucan (BG), the major molecular components of Gram negative bacteria and fungi, respectively^14^, and ii) chronic inflammation enhances lupus progression^15,16^, thus gut-translocation of these molecules might also have an impact upon lupus.

Thus, the influence of gut-leakage and/or the alteration of gut-microbiota were tested in asymptomatic lupus mice of FcGRIIb−/− (genetic cause) and pristane (environmental induction). Gut-leakage due to asymptomatic colitis was induced by 60 days of the low dose dextran sulfate solution (DSS)^17,18^ and the alteration of gut-microbiota was performed by co-housing (together with fecal gavage) with 40-wk-old FcGRIIb−/− mice (symptomatic lupus mice) or age-matched WT control. In addition, the impact of DSS upon lupus severity was also examined in symptomatic lupus mice at 24-wk-old.

## Materials and Methods

### Animal and the models of lupus

All experimental methods for animal care and use were performed and approved by the Institutional Animal Experimentation Ethics Committee of the Faculty of Medicine, Chulalongkorn University under protocol number 022/2561 the year 2017 in accordance with the Guide for the Care and Use of Laboratory Animals (eight edition), National Research Council. Female mice were used in all experiments. FcGRIIb−/− mice (C57BL/6 background) were kindly provided by Dr. Silvia Bolland (NIAID, NIH, Maryland, USA) and WT mice were purchased from the National Laboratory Animal Center(Nakornpathom, Thailand). Mice, housed in standard clear plastic cages (3–5 mice per cage), had free access to water and food with a light/dark cycle of 12: 12 h in 22 ± 2 °C with 50 ± 10% relative humidity. Pristane injection was used to induce lupus in WT following a previous publication^7^. Survival analysis of FcGRIIb−/− and pristane mice was performed by housing 5 female mice per cage for 15 months before sacrifice. Since lupus characteristics demonstrated as early as 20 wks post-pristane injection^7^ and in 24-wk-old FcGRIIb−/− mice^5,19^, pristane (Sigma-aldrich, St. Louis, MO, USA) (0.5 ml) was administered in 4-wk-old WT mice to induce lupus approximately at 24-wk-old to match with FcGRIIb−/− mice. Due to age-dependent lupus characteristics in both lupus models, mice at 8- and 24-wk-old were used as representatives of asymptomatic and symptomatic lupus, respectively. All mice at 8- and 24-wk-old were tested for lupus characteristics including serum creatinine (Cr), spot urine protein creatinine index (UPCI; detail later) and serum anti-dsDNA immunoglobulin (Ig) before the further experiments. Symptomatic lupus was defined as increased serum anti-dsDNA Ig together with high level of UPCI and/or serum Cr in comparison with age-matched control WT mice.

### Dextran sulfate solution (DSS) induced gut-leakage and co-housing with fecal gavage for gut microbiota alteration

**Figure 1:**
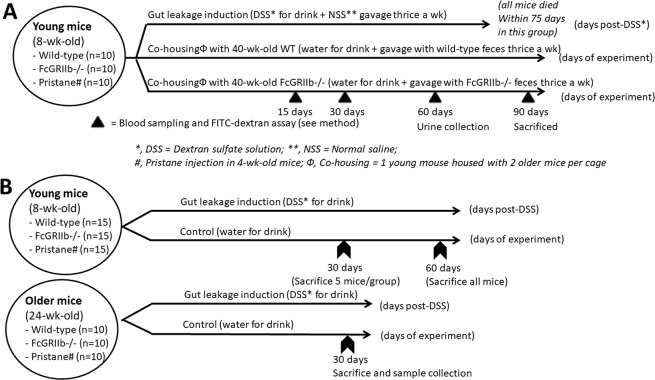
Schematic diagrams of the experiment for survival analysis and gut permeability determination (**A**), exploration of the influence of dextran sulfate solution (DSS) induced gut-leakage in 8- and 24-wk-old mice (**B**) were demonstrated.

**Figure 2:**
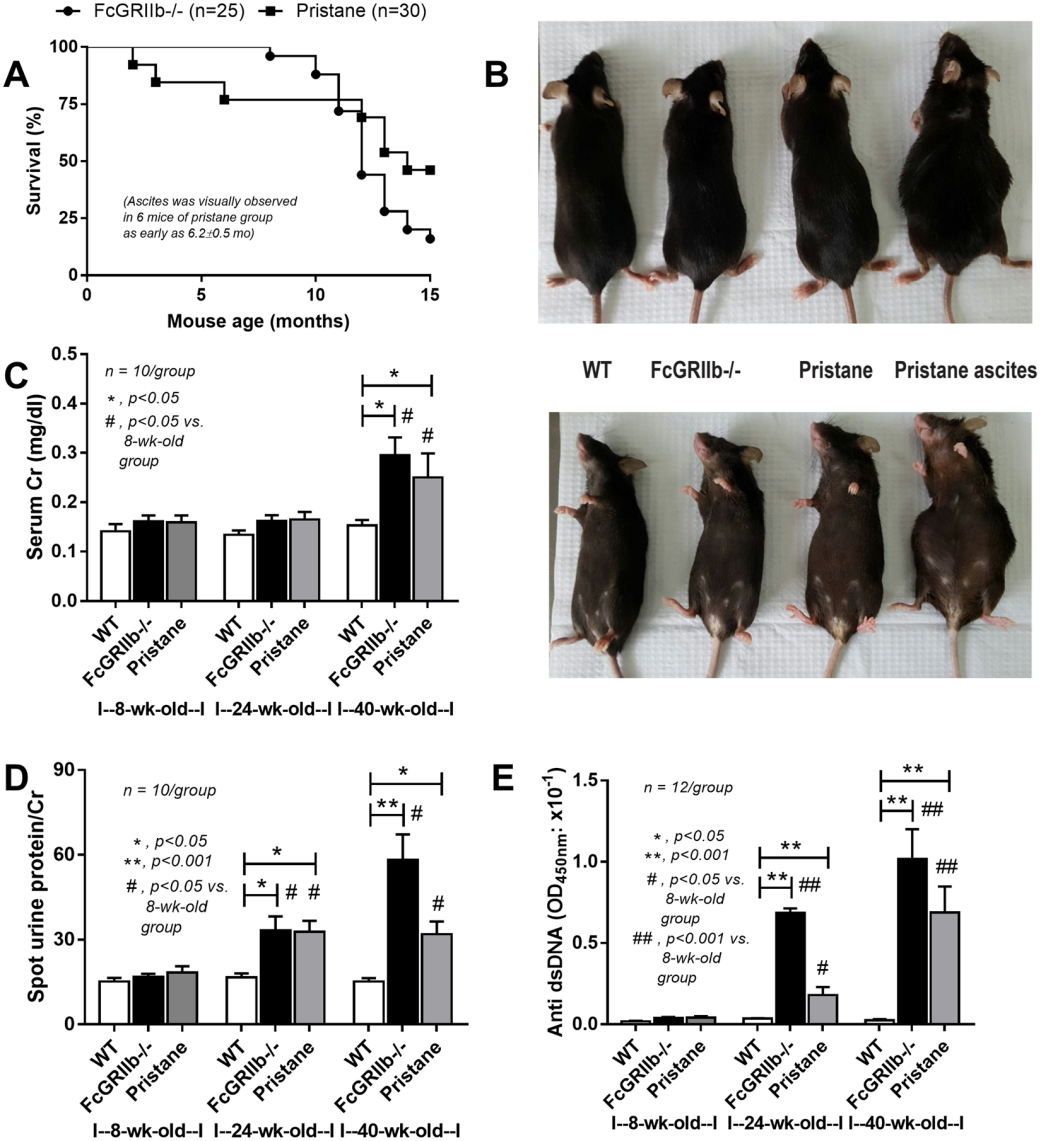
Survival analysis of FcGRIIb−/− and pristane mice (**A**), representative picture of visual characteristics of ascites in pristane mice (all 40-wk-old mice) (**B**) and representative lupus characteristics including serum creatinine (Cr), spot urine creatinine index (UPCI) and serum anti-dsDNA Ig in 8- and 24-wk-old mice in different groups (**C**–**E**) were demonstrated.

Dextran sulfate solution (DSS) (Sigma-Aldrich, St. Louis, MO, USA) at concentrations of 1% (w/v) was replaced for drinking water in FcGRIIb−/− and pristane mice at 8- and 24-wk-old to evaluate the influence of gastrointestinal-barrier defect (gut-leakage) upon asymptomatic and symptomatic lupus, respectively, and also in age-matched WT. Asymptomatic subtle colitis in mice with 8 wks of low dose DSS was previously described^17^. In parallel, co-housing with fecal gavage was used to test the influence of gut microbiota alteration against lupus progression. For the co-housing procedure, a younger mouse was co-housed with two 40-wk-old FcGRIIb−/− mice in 1 cage (3 mice per cage). Another set of 40-wk-old FcGRIIb−/− mice (5 mice per cage) were used as fecal donors for gavage three times a week into the younger co-housed mice to ensure allocoprophagy (the consumption of feces from other mice). FcGRIIb−/− fecal donor mice were separated in metabolic cages (Hatteras Instrument) for a few hours to collect feces. Fresh feces from 5 mice from different cages (housed 2 mice per cage and total 10 fecal donor mice were prepared) were mixed, diluted in phosphate buffer solution (PBS; 0.6 g feces in 1 ml PBS) and orally administered in mice at 20 ml/kg/dose. All DSS and fecal gavage mice were sacrificed after 90 days of the experiments with cardiac puncture under isoflurane anesthesia. Schematic diagrams of experiments are demonstrated in Fig. 1 with initially 10 mice in each group. Blood sample collection through facial artery for anti-dsDNA Ig was performed at 1 day prior to gut permeability determination (detail later) in specific time-point (Fig. 1A). Pristane mice at 40-wk-old were not used as fecal donors because of the possibility of difference in fecal microbiota as demonstrated in pristane with and without ascites (Fig. 2B). In addition, the influence of DSS induced gut-leakage was further tested in 8-wk-old mice (for 60 days) and 24-wk-old mice (for 30 days) with initial 10 mice in each group (WT, FcGRIIb−/− and pristane) (Fig. 1B). Of note, the duration of DSS administration in 24-wk-old mice was shorter than the administration in 8-wk-old mice due to the tendency of a higher mortality rate.

### Gut permeability test

Gut permeability was determined by the detection of fluorescein isothiocyanate-dextran (FITC-dextran), a non-absorbable molecule through the intestine, in serum after oral administration^18^. Briefly, 0.5 ml of FITC-dextran (molecular weight 4.4 kDa; FD4; Sigma, St. Louis, MO, USA) at a concentration of 25 mg/ml in sterile phosphate buffer solution (PBS) was orally administered and collected blood through tail vein at 3 h later. Serum FITC-dextran was measured by fluorospectrometry (NanoDrop 3300; Thermo Scientific, Wilmington, DE). In addition, leaky-gut was also determined by the spontaneous increased endotoxin (LPS) and (1→3)-β-D-glucan (BG) in serum. Serum LPS and BG was analyzed with HEK-Blue LPS Detection (InvivoGen, San Diego, CA, USA) and Fungitell assay (Associates of Cape Cod, East Falmouth, MA, USA), respectively. Values of LPS < 0.01 EU/mL and BG <7.8 pg/mL were recorded as 0.

### Serum sample analysis

Blood collection was performed through tail vein or cardiac puncture. Serum anti-dsDNA Ig in mouse was examined following a previously published protocol using Calf DNA (Invitrogen, Carlsbad, CA, USA) coated on 96-well plates^20^. Serum creatinine (Scr) was determined by QuantiChrom Creatinine-Assay (DICT-500, BioAssay, Hayward, CA, USA). Spot urine protein creatinine index (UPCI) followed the equation; UPCI = urine protein (mg/dl)/ urine creatinine (mg/dl) was used for the representative of proteinuria. Urine protein and creatinine was measured by Bradford protein assay and QuantiChrom Creatinine assay, respectively. Serum cytokines (IL-6), a representative of systemic inflammatory response, was measured by ELISA (ReproTech, Oldwick, NJ, USA). Serum LPS and BG were measured as previously mentioned.

### Histology, immunohistochemistry and flow cytometer analysis

Kidney (4 mm) was fixed in 10% formalin, paraffin embedded, and stained with Periodic acid–Schiff (PAS) stain (Sigma-Aldrich) for the semi-quantitative evaluation^21,22^. Glomerular injury was determined by percentage of moderate-severe glomerular injury (mesangial expansion >50%, crescentic formation and/or glomerulosclerosis) at 400x magnification and interstitial injury was semi-quantitatively estimated at 200x magnification using 10 randomly selected fields by the criteria of damage-area (cell infiltration, interstitial edema and tubular injuries) as following: 0, <5% area; 1, 5–10% area; 2, 10–25% area; 3, 25–50% area; and 4, >50% area. The immune complex deposition in glomeruli was visualized by immunofluorescence prepared in Cryogel (Leica Biosystems, Richmond, IL, USA), stained with goat anti-mouse IgG (Alexa Fluor 488, Abcam, Cambridge, MA, USA) and detected by ZEISS LSM 800 (Carl Zeiss, Germany). Spleen apoptosis was detected by immunohistochemistry with anti-active caspase 3 antibody (Cell Signaling Technology, Beverly, MA, USA) (expressed as positive cells per high-power field)^22^ and flow cytometry. The fluorochrome-conjugated antibodies against different molecules were used including; i) apoptosis indicators, annexin V and propidium iodide (PI), ii) B220 (B cell) and iii) F4/80 (macrophage) (BioLegend, San Diego, CA, USA) with FlowJo software^23^.

### Mesenteric lymph node analysis

DSS-induced gut bacterial translocation is determined by bacterial culture positive in mesenteric lymph node (MLN) despite the absence of bacteremia^24^. Also, MLN (delivering to thoracic duct) and portal vein (carrying to liver) are the important routes of the translocation from gut into blood circulation^25^. At sacrifice, MLNs (3–6 nodes) were homogenized, sonicated in PBS in different dilutions, directly streaked onto blood agar plates (Oxoid, Hampshire, UK) and incubated at 37 °C for 24 h before bacterial colony enumeration. In parallel, apoptosis in MLN was explored by flow cytometry analysis and activated caspase 3 as previously described.

### Macrophage and starvation induced cell-injuries

Macrophages, derived from bone marrow (BM) following a published protocol^26^, were used to test the impact of LPS (and/or BG). Macrophages (1 × 10^5^ cells/well) were incubated with Pachyman (a representative BG; at 10 µg/ml) with or without LPS (*Escherichia coli* 026:B6; Sigma-Aldrich; at 10 ng/ml) for 24 h before supernatant collection, apoptosis detection or further starvation. The apoptosis was detected by FITC-annexin V apoptosis detection assay (BioLegend) with flow cytometry (BD FACSVia™ system) using Flowjo software.

To test the cell-vulnerability to injury after the stimuli (LPS and/or BG), cell starvation was induced by Earle’s balanced salt solution (Gibco, Grand Island, NY, USA) for 4 h at 37 °C in 5% CO_2_. After that, several cell injury parameters were determined following previous publications^27,28^ including; (i) TNF-related apoptosis inducing ligand (TRAIL), (ii) mitochondrial DNA (mtDNA), iii) cellular reactive oxygen species (ROS) production and iv) cellular ATP content. In short, TRAIL was evaluated by QuantStudio® 6 Real-Time PCR system (Applied Biosystems, Life Technology Corporation, CA, USA) using *RNeasy* and cDNA synthesis Kit (Qiagen, Albertslund, Denmark) with micro-β-actin as a comparative endogenous control by ∆Ct method^27^. Also, mtDNA was measured by FavorPrep™ Tissue Genomic DNA Extraction assay (Favorgen Biotech corp, Wembley, WA, Australia) using primers of mitochondrial encoded mtDNA (mMito F, mMito R) and nuclear encoded β-2-microglobulin (mβ2m F, mβ2m R) on QuantStudio® 6 Real-Time PCR system (Applied Biosystems)^28^. The relative abundance of nuclear and mitochondrial DNA was evaluated in terms of fold change against the untreated WT macrophage by 2-∆∆CT method. Real time RT-PCR was performed with Mastermix 1xKAPA fast SYBR Green (Kapa Biosystems, Wilmington, MA, USA) and 2 µL of DNA template. In addition, ROS production (oxidative stress) and ATP content was determined by oxidative fluorescent dye Dihydroethidium (DHE) (Sigma-Aldrich) and Luminescent ATP Detection Assay (Abcam), respectively, before visualized with Varioskan Flash microplate reader (Thermo-Scientific)^28^.

### Statistical analysis

Data were presented in mean ± standard error (SE) and statistical differences among groups were examined using unpaired Student’s t-test or one-way analysis of variance (ANOVA) with Tukey’s comparison test for the analysis of experiments with 2 and 3 groups, respectively. Data with several time-points were conducted by repeated-measures ANOVA with Bonferroni post-hoc analysis. Survival analyses were evaluated with the log-rank test. *P* values < 0.05 were considered statistically significant. SPSS 11.5 software (SPSS, Chicago, IL, USA) was used for all statistical analyses.

## Results

The similarity between lupus models (without the experimental interventions) of FcGRIIb−/− mice and pristane induction was demonstrated by non-significant differences in survival analysis, serum creatinine (Cr) and proteinuria, although 6 out of 30 pristane mice developed ascites by visual observation as early as 6.2 ± 0.5 wk old (Fig. 2). FcGRIIb−/− mice demonstrated a tendency of the higher model severity (Fig. 2A–E).

### Gastrointestinal leakage enhanced lupus disease progression in FcGRIIb deficient and pristane mice

**Figure 3:**
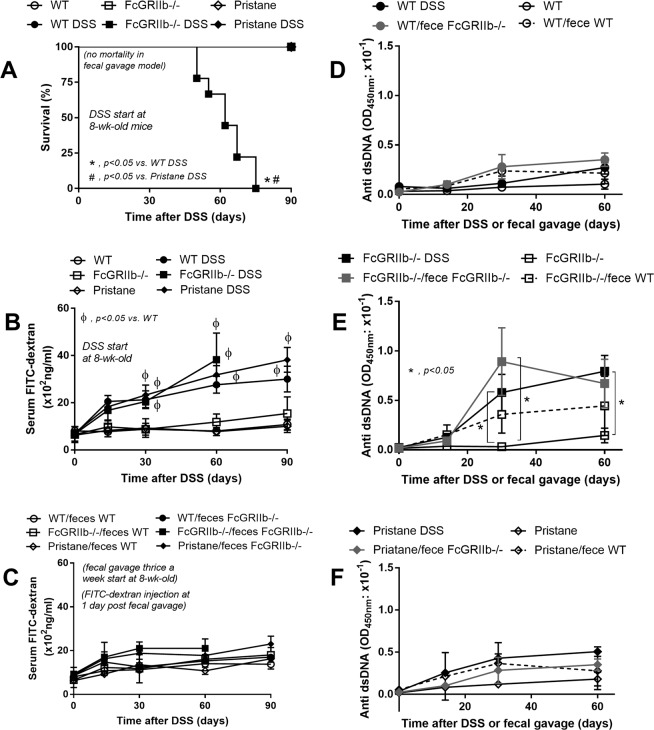
The survival analyses of mice with and without gut-leakage induced by dextran sulfate solution (DSS) in drinking water or control water of wild-type (WT), FcGRIIb−/− and pristane mice (**A**) (n = 10 per group) and time course of gut-leakage as measured by FITC-dextran assay of DSS mice (**B**) and mice in the protocol of co-housing (plus fecal gavage) with 40-wk-old WT (WT/ feces WT) or 40-wk-old FcGRIIb−/− (WT/feces FcGRIIb−/−) (**C**) (n = 6–9 per time-point) were demonstrated. In addition, time-course of serum anti-dsDNA Ig in mice with DSS or the co-housing (plus fecal gavage) in WT, FcGRIIb−/− and pristane mice (**D**–**F**) (n = 6–9 per time-point) were demonstrated.

The influence of leaky-gut (DSS) or gut microbiota (co-housing with fecal gavage) against lupus progression was tested in 8-wk-old mice. All of FcGRIIb−/− mice died within 75 days post-DSS administration (Fig. 3A) without diarrhea or bacteremia (data not shown) while there was no mortality in mice with fecal gavage (Fig. 3A). All pristane mice with DSS also survived. DSS, but not fecal gavage, induced leaky-gut as demonstrated by FITC-dextran assay (Fig. 3B,C). The severity of DSS-induced leaky-gut was similar between WT and lupus mice (Fig. 3B). Because FcGRIIb−/− mice with DSS might die from active lupus, anti-dsDNA Ig, a lupus autoimmune antibody, was explored. While DSS and fecal gavage did not enhance anti-dsDNA Ig in WT mice, DSS induced rapid increased anti-dsDNA Ig in FcGRIIb-/- (Fig. 3D,E). Fecal gavage also enhanced anti-dsDNA Ig in FcGRIIb−/−, but not in pristane (Fig. 3E,F), suggesting the influence of the gene-defect. Of note, the gavage of WT feces into FcGRIIb−/− mice showed a tendency of increased anti-dsDNA Ig but did not reach a statistically significant value (Fig. 3E).

**Figure 4:**
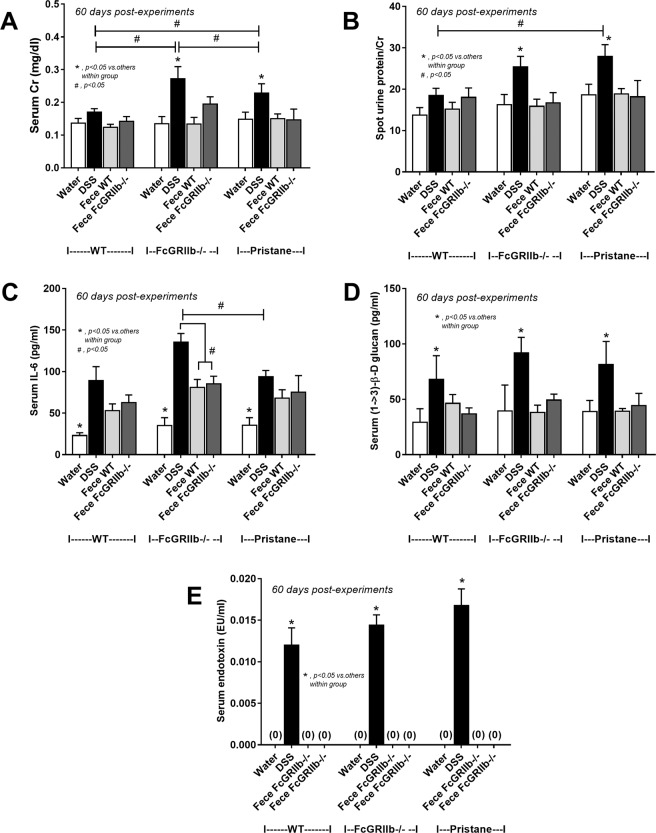
The characteristics of wild-type (WT), FcGRIIb−/− and pristane mice with 60 days of dextran sulfate solution (DSS) (started at 8-wk-old; asymptomatic lupus) or water control drinking water as determined by renal injury (serum creatinine; Cr) (**A**), proteinuria (spot urine/creatinine) (**B**), systemic inflammation (serum IL-6) (**C**), and leaky-gut as determined by spontaneous serum (1→3)-β-D-glucan (BG) (**D**) and spontaneous endotoxemia (**E**) were demonstrated. In addition, these characteristics of mice with the protocol of co-housing (plus fecal gavage) by feces of 40-wk-old WT or 40-wk-old FcGRIIb−/− (**F**–**J**) and the comparison between DSS versus the co-housing (plus fecal gavage) (**K**–**O**) were indicated (n = 7–10 per group). *p < 0.05, ^#^*p* < 0.05 vs. WT DSS.

**Figure 5:**
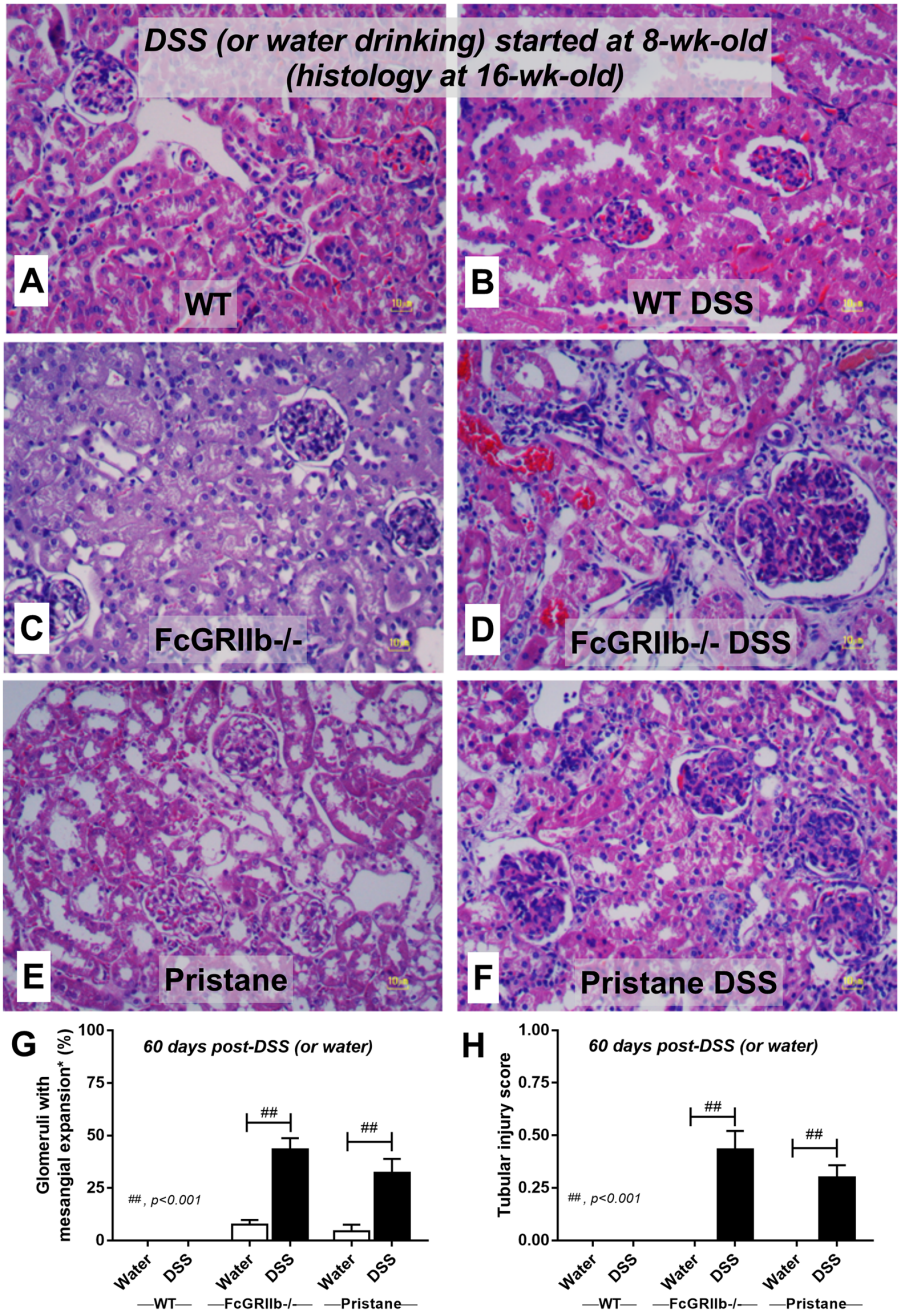
Representative renal histopathology by Periodic Acid–Schiff (PAS) staining of wild-type (WT), FcGRIIb−/− and pristane mice at 16-wk-old with or without 60 days of dextran sulfate solution (DSS) in drinking water (**A**–**F**) and the injury score from glomeruli (**G**) and tubular injury (**H**) (see methods) were demonstrated (n = 4 per group). Glomerular hypertrophy were noted in FcGRIIb−/− and pristane with DSS.

**Figure 6:**
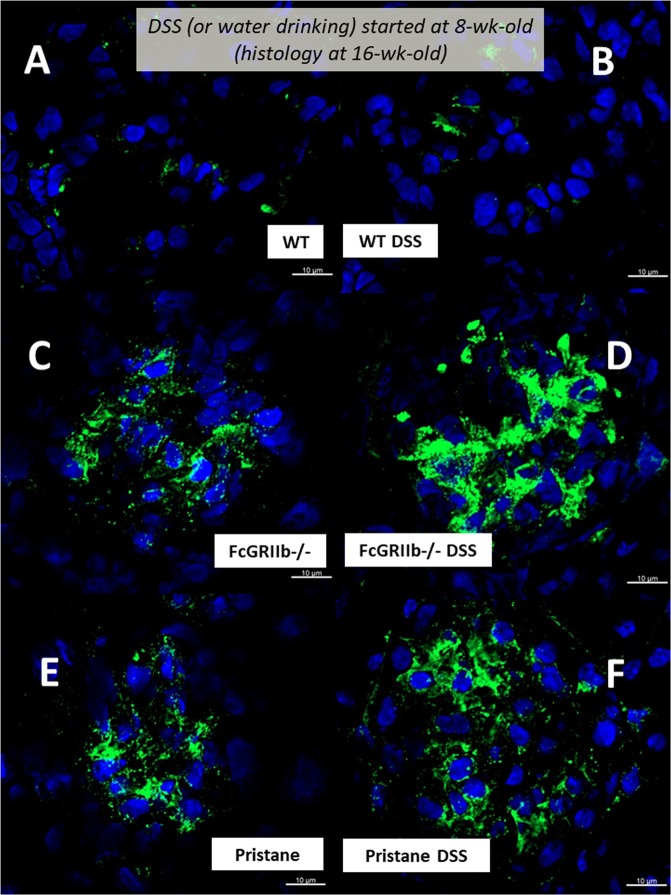
Representative immunofluorescence pictures from glomeruli of wild-type (WT), FcGRIIb−/− and pristane mice at 16-wk-old with or without 60 days of dextran sulfate solution (DSS) in drinking water (**A**–**F**) were demonstrated.

**Figure 7:**
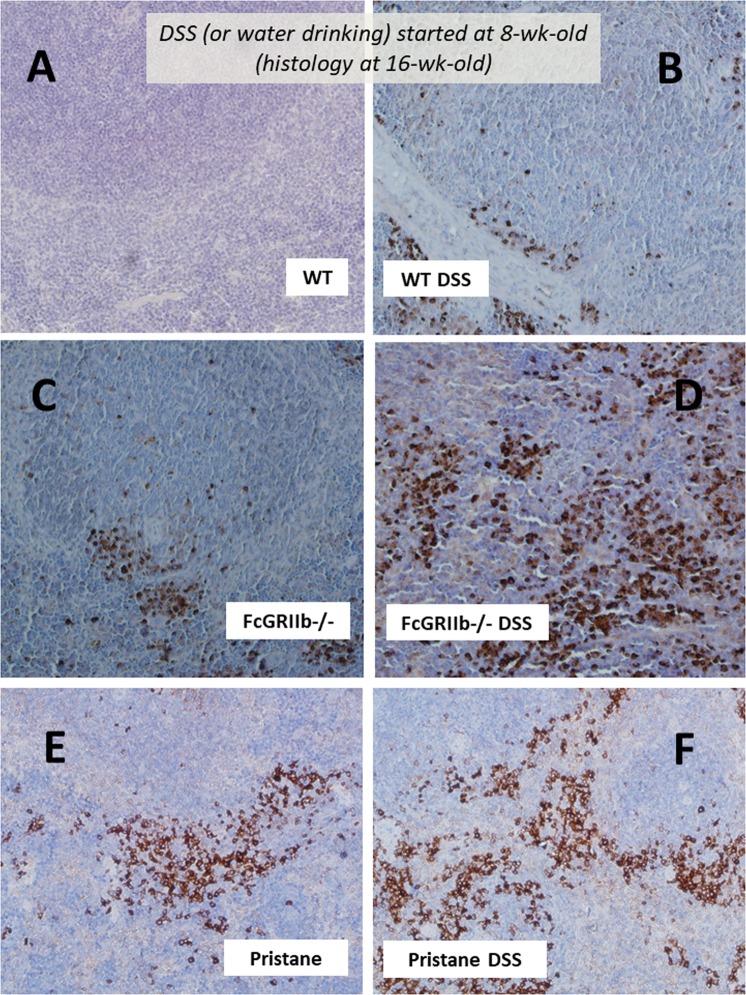
Representative immunohistochemistry of apoptosis detection by active caspase-3 from spleen of wild-type (WT), FcGRIIb−/− and pristane mice at 16-wk-old with or without 60 days of dextran sulfate solution (DSS) in drinking water (**A**–**F**) were demonstrated.

In parallel, DSS-induced leaky-gut enhanced renal injury (serum Cr, proteinuria, histology and immune complex deposition in glomeruli) in lupus mice (both pristane and FcGRIIb−/−) but not WT (Figs. 4A,B, 5–7). Likewise, DSS increased systemic inflammatory responses (serum IL-6) together with spontaneous presentation of endotoxin and (1→3)-β-D-glucan (BG) in serum, leaky-gut indicators^18,29^, in both WT and lupus mice (Fig. 4B–E). The similar blood level of FITC-dextran (Fig. 3B), LPS and BG (Fig. 4D,E) between WT and lupus models with DSS implies a similar injury severity from DSS. Of note, serum Cr and serum IL-6 in FcGRIIb−/− mice were higher than pristane mice after DSS induction (Fig. 4A,C). In contrast, co-housing with fecal gavage could not induce leaky-gut, renal injury and proteinuria in all groups (Fig. 4).

### Gastrointestinal leakage, endotoxin and (1→3)-β-D-glucan versus apoptosis (in spleen and mesenteric lymph node) of lupus mice

**Figure 8:**
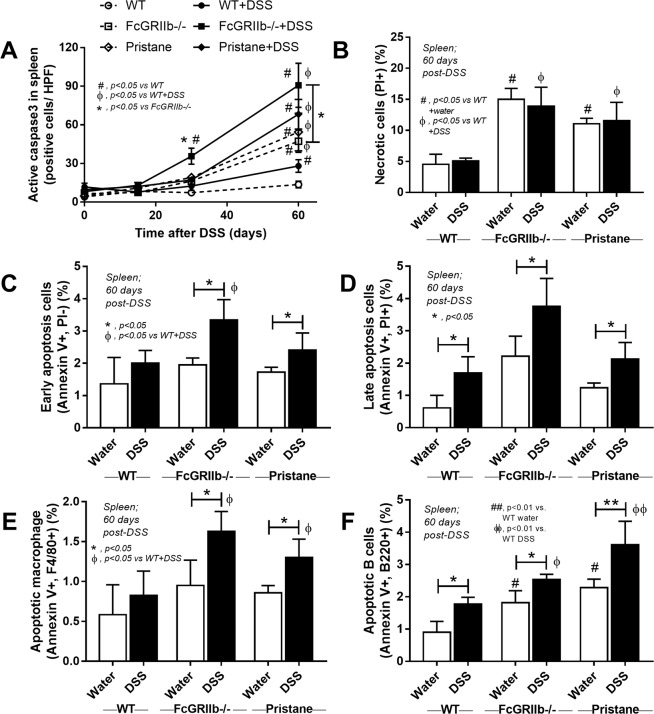
Semi-quantitative analysis score of active caspase-3 in spleen of wild-type (WT), FcGRIIb−/− and pristane mice with or without 60 days of dextran sulfate solution (DSS) in drinking water (**A**) were demonstrated. In addition, the quantitative flow-cytometric analysis of splenocyte from these mice in term of necrotic cells (propidium iodide, PI +ve) (**B**), early apoptosis cells (Annexin V +ve, PI −ve) (**C**), late apoptosis cells (Annexin V +ve, PI +ve) (**D**), apoptotic macrophage in spleen (Annexin V +ve, F4/80 +ve) (**E**) and apoptotic B cell in spleen (Annexin V +ve, B220 +ve) were indicated (n = 4–6 per group).

Apoptosis induces anti-dsDNA Ig and deteriorates lupus progression^30^. As such, spleen apoptosis determined by caspase 3 staining was detectable as early as 30 days post-DSS in FcGRIIb−/− mice (Figs. 7, 8A) which possibly associated with high anti-dsDNA Ig at 30 and 60 days post-DSS (Fig. 3E). At 60 days post-experiment, apoptosis in spleen was detectable in lupus mice (FcGRIIb−/− and pristane) with or without DSS administration and also in WT with DSS (Fig. 8A and Supplementary Fig. S1). Despite the spontaneous spleen apoptosis in FcGRIIb−/− mice, DSS enhanced apotosis severity (Fig. 8A). Of note, the severity of spleen apoptosis at 60 days post-DSS as examined by active caspase-3 staining and flow-cytometric analysis was similar between FcGRIIb−/− and pristane (Fig. 8B–F). DSS also induced late apoptosis in WT spleen, but less severe than lupus (Fig. 8D), indicating the influence of lupus in apoptosis susceptibility. The apoptotic splenocytes after DSS induction were identified as B-cell (both in lupus and WT) and macrophage (only in lupus but not WT) (Fig. 8E,F).

**Figure 9:**
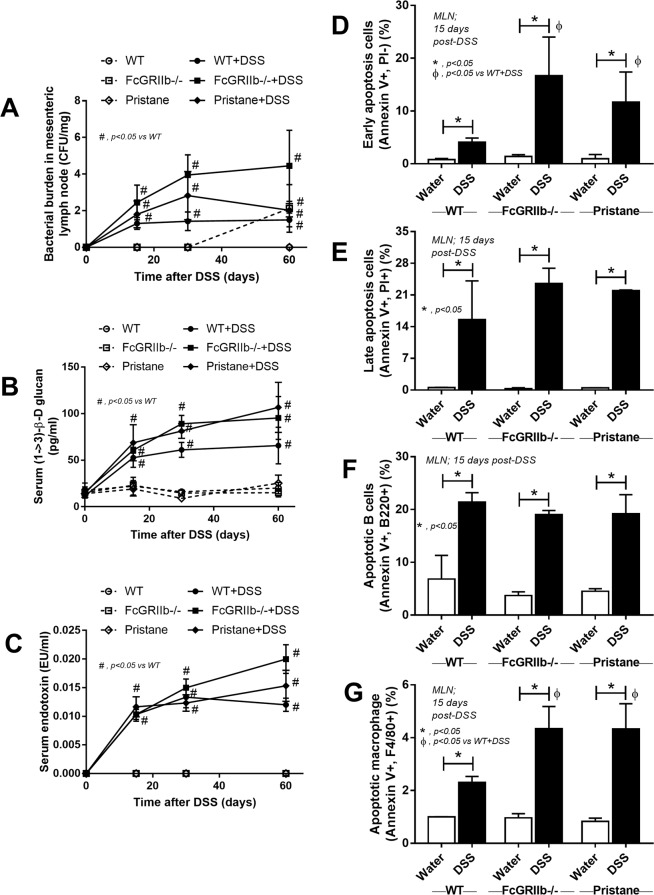
Bacterial burdens in mesenteric lymph node (MLN) in wild-type (WT), FcGRIIb−/− and pristane mice with or without of dextran sulfate solution (DSS) in drinking water at different time-points (**A**), endotoxemia and serum (1→3)-β-D-glucan (BG) (**B**,**C**) were demonstrated (n = 5–6 per time-point for **A**–**C**). In addition, early apoptosis cells (Annexin V +ve, PI −ve), late apoptosis cells (Annexin V +ve, PI +ve), B cell apoptosis (Annexin V +ve, B220 +ve) and macrophage apoptosis (Annexin V +ve, F4/80 +ve) in MLN (**D**,**E**) were demonstrated (n = 5–6 per group for **D**,**E**).

**Figure 10:**
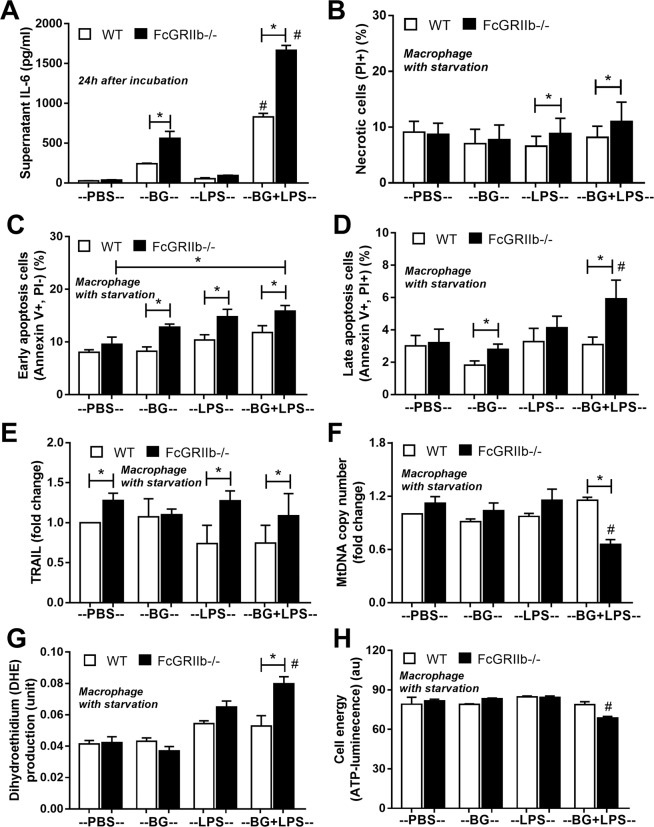
The immune response of macrophage from wild-type (WT) and FcGRIIb−/− after stimulated *in vitro* with phosphate buffer solution control (PBS) or LPS with and without purified (1→3)-β-D-glucan (BG) after 24 h incubation was demonstrated (**A**). Quantitative flow-cytometric analysis of macrophage from WT and FcGRIIb−/− mice after LPS-stimulation with and without BG following by cell starvation (see methods) to determine necrotic cells (propidium iodide; PI +ve) (**B**), early apoptosis cells (Annexin V +ve, PI −ve) (**C**), late apoptosis cells (Annexin V +ve, PI +ve) (D) were demonstrated. In addition, other parameters of macrophage injury from these activations in TNF-related apoptosis-inducing ligand (TRAIL) (**E**), copy numbers of mitochondria DNA (mtDNA) (**F**), reactive oxygen species production as detected by dihydroethidium (DHE) (**G**) and total cell energy with ATP luminescence intensity (**H**) were indicated (independent triplicate experiments were performed). *p < 0.05; ^#^*p* < 0.05 vs. same mouse strain in other experimental groups.

In parallel, DSS-induced gut-leakage was also supported by the detection of bacteria in mesenteric lymph node (MLN) and the spontaneous elevation of endotoxin and BG in serum without systemic infection (Fig. 9A–C). Also, DSS induced apoptosis in MLN (Fig. 9D–G and Supplementary Fig. S2) in a similar characteristic to spleen (Fig. 8) possibly due to the impact of LPS^31–33^ and BG^34^. Indeed, the additional effect of BG on top of LPS was demonstrated by IL-6 induction in macrophage from both mouse strains (predominantly in FcGRIIb−/− cell), as a test of concept (Fig. 10A), but could not directly activate apoptosis (data not shown). Because *in vivo* macrophages are stimulated by several stresses, cell starvation is used as a representative. As such, macrophage pre-conditioning with LPS plus BG was susceptible to starvation-induced apoptosis (predominantly on FcGRIIb−/− cell) (Fig. 10B–D), possibly due to the reduced cell-energy (mitochondria and ATP) and increased reactive oxygen species (DHE), but not from the activation by TRAIL (a well-known apoptosis activated molecule) (Fig. 10E–H and Supplementary Fig. S3).

### Gastrointestinal leakage enhanced lupus disease-severity in symptomatic lupus mice (FcGRIIb deficient and pristane)

**Figure 11:**
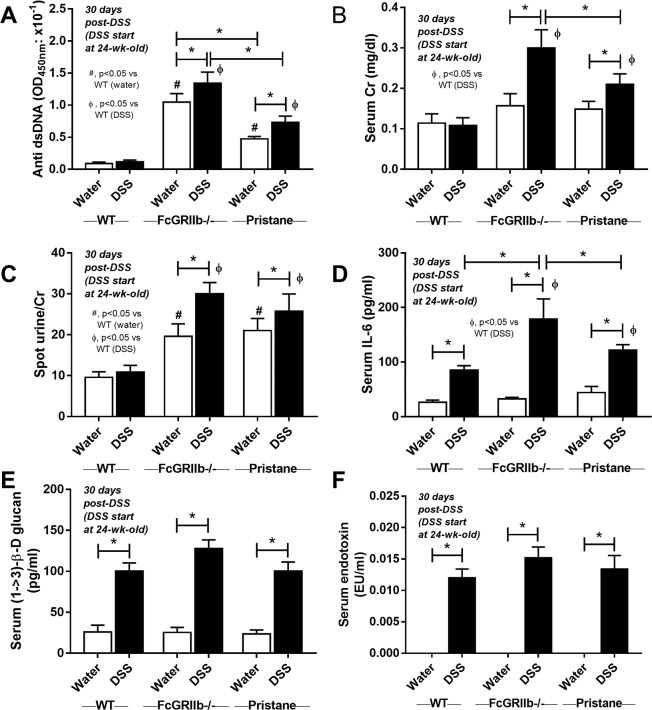
The characteristics of wild-type (WT), FcGRIIb−/− and pristane mice with 30 days of dextran sulfate solution (DSS) or water control drinking water (started at 24-wk-old; symptomatic lupus) as determined by serum anti-dsDNA Ig (**A**), renal injury (serum creatinine; Cr) (**B**), proteinuria (spot urine/creatinine) (**C**), systemic inflammation (serum IL-6) (**D**), and leaky-gut as determined by spontaneous serum (1→3)-β-D-glucan (BG) (**E**) and spontaneous endotoxemia (**F**) were demonstrated (n = 7–10 per group). *p < 0.05, ^#^*p* < 0.05 vs. WT DSS.

**Figure 12:**
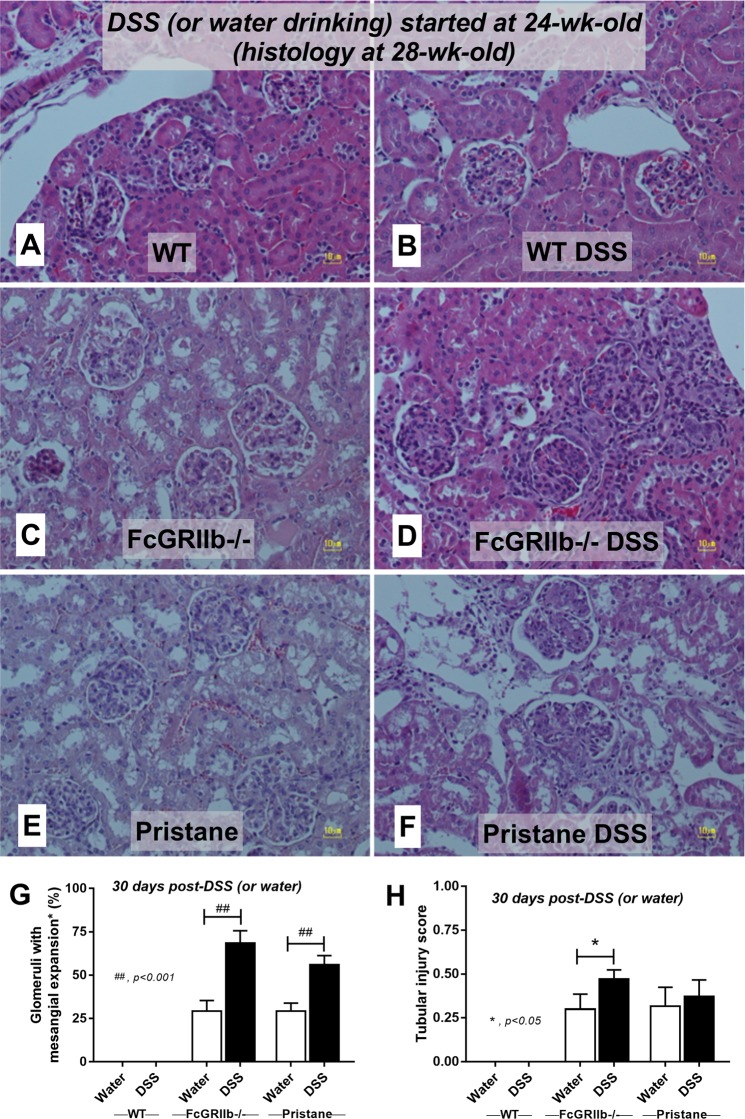
Representative renal histopathology by Periodic Acid–Schiff (PAS) staining of 28-wk-old of symptomatic lupus (FcGRIIb−/− and pristane) and age-matched wild-type (WT) with or without 30 days of dextran sulfate solution (DSS) in drinking water (**A–F**) and the injury score from glomeruli (**G**) and tubular injury (**H**) (see methods) were demonstrated.

**Figure 13:**
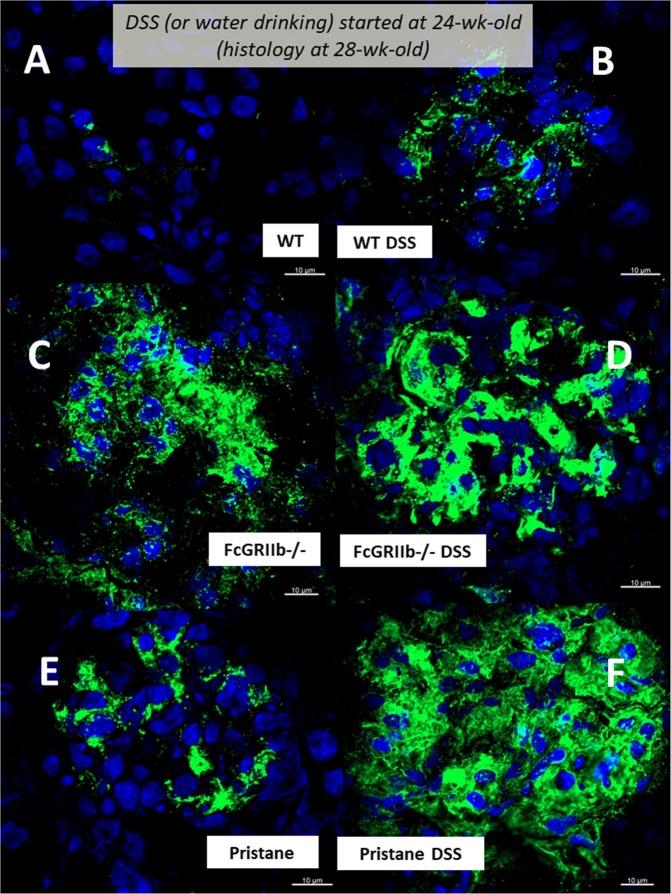
Representative immunofluorescence pictures from glomeruli of 28-wk-old mice of symptomatic lupus (FcGRIIb−/− and pristane) and age-matched WT (WT) with or without 30 days of dextran sulfate solution (DSS) in drinking water (**A**–**F**) were demonstrated.

Because FcGRIIb−/− mice developed proteinuria as early as 24-wk-old^23^, DSS was administered in 24-wk-old FcGRIIb−/− (and pristane) mice to explore the influence of leaky-gut in symptomatic lupus condition. At 30 days post-DSS, anti-dsDNA Ig, serum Cr and serum IL-6 in FcGRIIb−/− mice was higher than pristane (Figs. 11–13) despite the similar level of LPS and BG in blood (Fig. 4D,E) emphasizing the hyper-immune responses due to the inhibitory-signaling defect of FcGRIIb−/− group^3,23^. However, proteinuria, histopathology score and immunoglobulin deposition in glomeruli were not different between FcGRIIb−/− and pristane mice (Figs. 11–13).

## Discussion

DSS-induced gut-leakage facilitated the translocation of organism molecules from gut into blood circulation leading to the enhanced systemic inflammation, apoptosis induction and increased lupus progression in FcGRIIb−/− mice. These data support the potential impact of gut-leakage toward disease progression of lupus.

### Gut-leakage enhanced lupus disease progression in lupus mice

Spontaneous leaky-gut could not be demonstrated in 8-wk-old mice but DSS similarly enhanced gut-leakage in these mice  (both WT and lupus mice). Interestingly, DSS administration in asymptomatic young lupus mice, but not the manipulation on gut microbiota by the co-housing^35^, deteriorated lupus disease-severity (serum Cr and proteinuria) and increased mortality, implying the importance of leaky-gut upon lupus. Although the co-housing enhanced systemic inflammation and anti-dsDNA Ig in FcGRIIb-/- mice, it was not enough to induce other lupus manifestations. A longer period of gut-microbiota alteration might be necessary to demonstrate the clinical impact of the co-housing.

Because Gram negative bacteria and fungi are prominent constituents of the normal microbiota in gut^14,36^, gut-leakage induces the elevation of endotoxin and BG (foreign molecules in mammals) in serum. In addition, the detection of bacteria in MLN^24^ also supports DSS-induced gut-permeability barrier defect. Despite the similar severity of gut-leakage between FcGRIIb−/− and WT mice as determined by FITC-dextran assay, serum LPS, serum BG and bacterial burdens in MLN, gut-leakage demonstrated very less impact in WT mice, but worsen the disease-severity in lupus mice. Perhaps, early bacterial translocation in MLN induces cell apoptosis in MLN (15 days post-DSS) that is responsible for the increased production of anti-dsDNA Ig (30 days post-DSS) in lupus mice. In contrast, WT mice do not produce anti-dsDNA Ig against auto-antigens of apoptotic body different from the lupus mice^5,37,38^.

On the other hand, spleen apoptosis might cause by LPS and BG in serum supporting the reports on LPS-induced apoptosis in other lupus models^31–33^. Furthermore, serum BG also enhances the impact of LPS upon lupus progression because BG administration alone could enhance lupus^39–41^ and the synergistic effect of BG upon LPS through TLR-4 and Dectin-1 are reported^42–45^. Hence, LPS and BG from gut translocation induce more prominent apoptosis and higher immune response reaction (anti-dsDNA Ig) in lupus mice in comparison with WT.

### Gut-leakage and the increased mortality rate in FcGRIIb−/− mice

The predominant systemic inflammation (serum IL-6) in lupus mice over WT is possibly due to the inhibitory signalling defect and hyper immune-responsiveness of FcGRIIb−/−^3^ and pristane mice^7,46,47^, respectively. Although DSS enhanced lupus characteristics (anti-dsDNA Ig, serum Cr, proteinuria, etc.) in both lupus models, DSS increased mortality rate only in FcGRIIb−/− mice, suggesting an impact of genetic-background. As such, FcGRIIb in hepatic sinusoids also contributes to immune complex clearance^48^ and FcGRIIb in endothelial cells associated with cardiovascular diseases^49^. Indeed, FcGRIIb−/− macrophages with LPS/BG pre-conditioning were vulnerable to starvation injury (mitochondria injury, ROS production and apoptosis) and possibly other stresses that might be responsible for the high mortality rate after DSS administration in FcGRIIb−/− mice.

**Figure 14:**
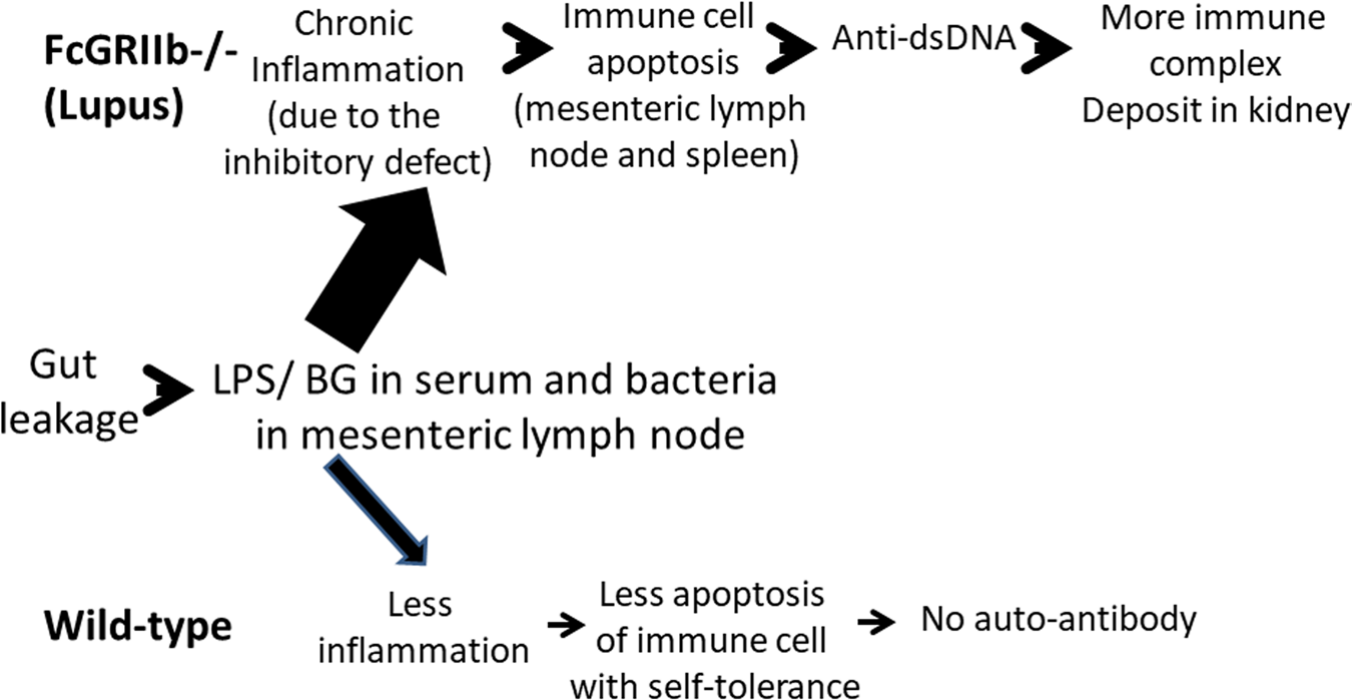
Proposed hypothesis of gut-leakage enhanced lupus progression. Gut-leakage induces the translocation of gut bacteria, endotoxin (LPS) and (1→3)-β-D-glucan (BG) either in FcGRIIb−/− mice or wild-type (WT). However, inflammatory responses, apoptosis of immune cells and anti-dsDNA Ig production are more prominent in FcGRIIb−/− lupus mice due to the loss of inhibitory signaling.

It is possible that LPS and BG in serum of lupus mice (from gut-translocation) might be a first hit on immune cells leading to cell-apoptosis, auto-antibody production, circulating immune complex (CIC) deposition and the disease-activity of lupus as concluded in Fig. 14. Nevertheless, the co-housing of asymptomatic lupus mice with the symptomatic lupus mice (with fecal gavage) also mildly activated serum IL-6 and anti-dsDNA Ig without increased gut-leakage. Hence, the small molecular products from gut microbiota that could pass through intact tight junctions might be responsible for the mild systemic response in this group. More mechanistic studies are worth exploring.

## Conclusions

Our data implied that (i) gut-leakage enhanced lupus progression and lupus disease activity, and (ii) organismal molecules from gut-translocation possibly worsens lupus through the induction of systemic inflammation, cell apoptosis, anti-dsDNA Ig production and CIC deposition. In translational aspects, strategies to attenuate or identify gut-leakage and/or elevated serum endotoxin (and BG) in patients with lupus might be beneficial.

## Supplementary information

Supplementary Information.

## Data Availability

All data generated and analyzed during the current study are available from the corresponding author on reasonable request.
