# Supplementary material for: Leaky-gut enhanced lupus progression in the Fc gamma receptor-IIb deficient and pristane-induced mouse models of lupus
Source: Sci Rep. 2020 Jan 21;10:777. doi: 10.1038/s41598-019-57275-0 (PMC6972921; doi:10.1038/s41598-019-57275-0)
Supplement: Supplementary file 1 — Supplementary Information. [file 41598_2019_57275_MOESM1_ESM.pdf]

Leaky-gut enhanced lupus progression in the Fc gamma receptor-IIb deficient and pristane-induced mouse models of lupus

Arthid Thim-uam, Saowapha Surawut, Jiraphorn Issara-Amphorn, Thiranut Jaroonwitchawan, Pratsanee Hiengrach, Piraya Chatthanathon, Alisa Wilantho, Naraporn Somboonna, Tanapat Palaga, Prapaporn Pisitkun, Asada Leelahavanichkul

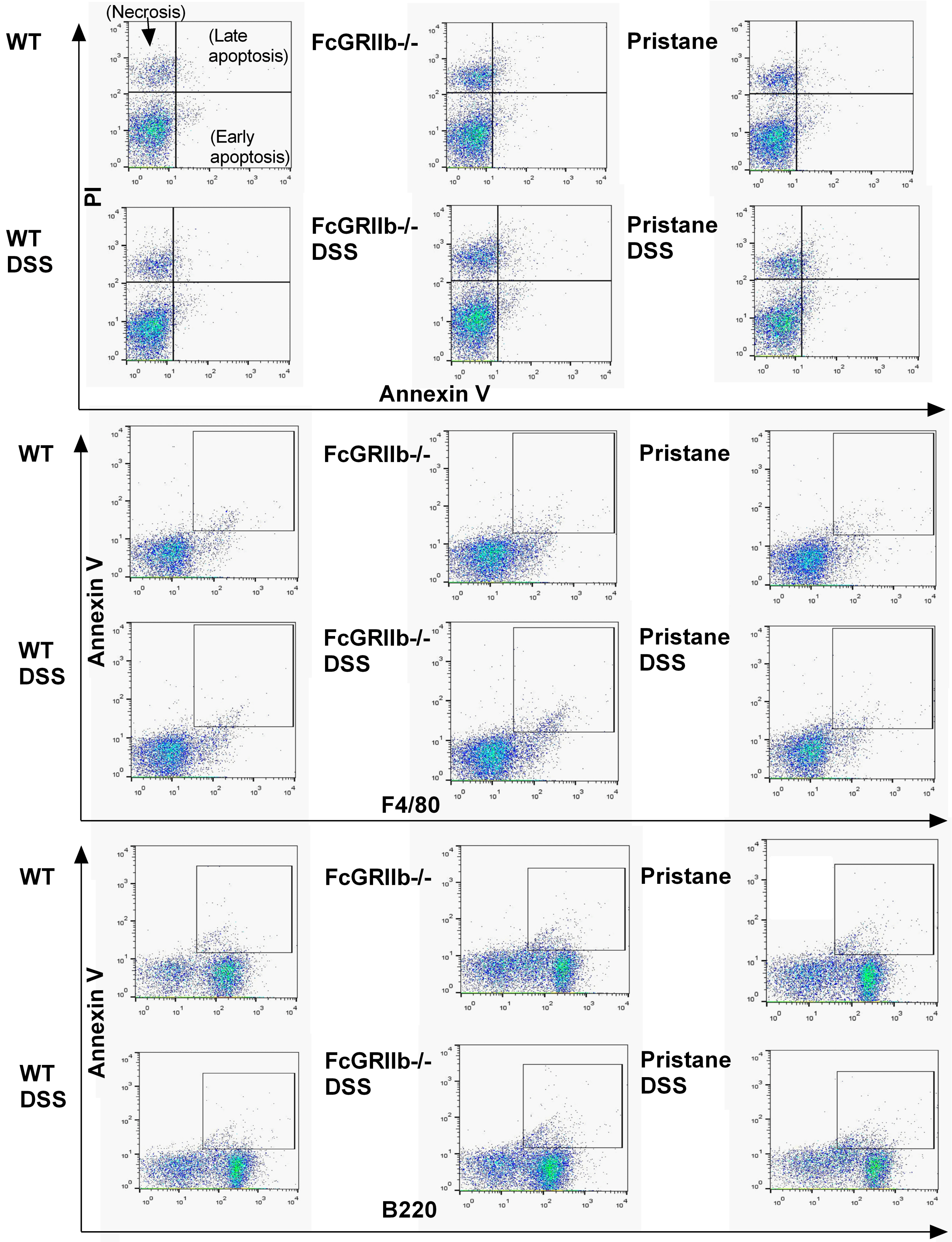

Supplementary Figure S1 Representatives of flow-cytometric analysis of splenocyte from WT, FcGRIIb-/- and pristane mice with and without DSS in necrotic cells (propidium iodide; PI +ve), early apoptosis cells (Annexin V +ve, PI –ve), late apoptosis cells (Annexin V +ve, PI +ve), apoptotic macrophage in spleen (Annexin V +ve, F4/80 +ve) and apoptotic B cell in spleen (Annexin V +ve, B220 +ve) were indicated (n = 4-6 per group).

Leaky-gut enhanced lupus progression in the Fc gamma receptor-IIb deficient and pristane-induced mouse models of lupus

Arthid Thim-uam, Saowapha Surawut, Jiraphorn Issara-Amphorn, Thiranut Jaroonwitchawan, Pratsanee Hiengrach, Piraya Chatthanathon, Alisa Wilantho, Naraporn Somboonna, Tanapat Palaga, Prapaporn Pisitkun, Asada Leelahavanichkul

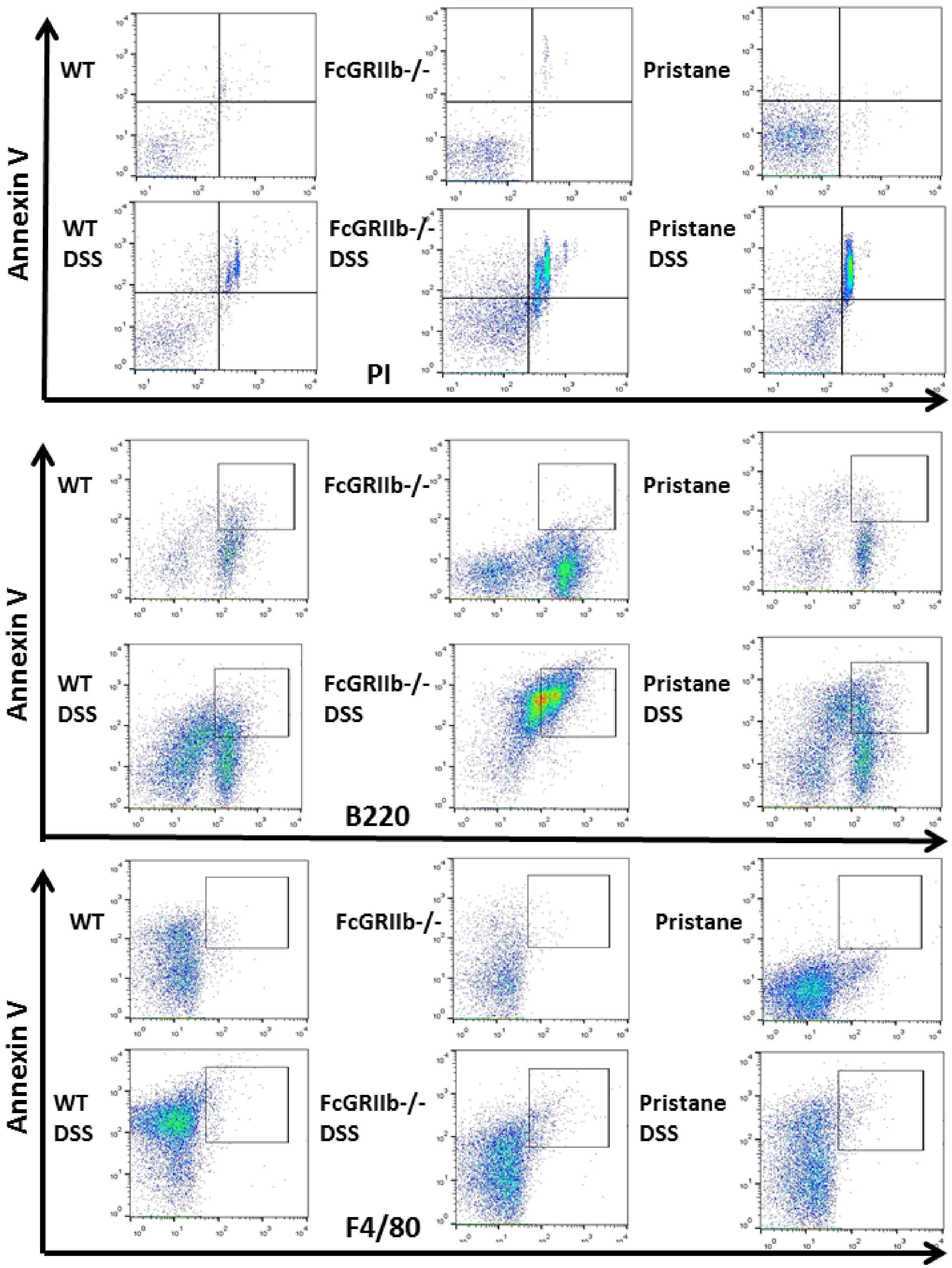

Supplementary Figure S2 Representatives of flow-cytometric analysis of mesenteric lymph node (MLN) from WT, FcGRIIb-/- and pristane mice with and without DSS as early as 15 days post-DSS in necrotic cells (propidium iodide; PI +ve) , early apoptosis cells (Annexin V +ve, PI –ve), late apoptosis cells (Annexin V +ve, PI +ve), apoptotic macrophage (Annexin V +ve, F4/80 +ve) and apoptotic B cell (Annexin V +ve, B220 +ve) were indicated (n = 4-6 per group).

Leaky-gut enhanced lupus progression in the Fc gamma receptor-IIb deficient and pristane-induced mouse models of lupus

Arthid Thim-uam, Saowapha Surawut, Jiraphorn Issara-Amphorn, Thiranut Jaroonwitchawan, Pratsanee Hiengrach, Piraya Chatthanathon, Alisa Wilantho, Naraporn Somboonna, Tanapat Palaga, Prapaporn Pisitkun, Asada Leelahavanichkul

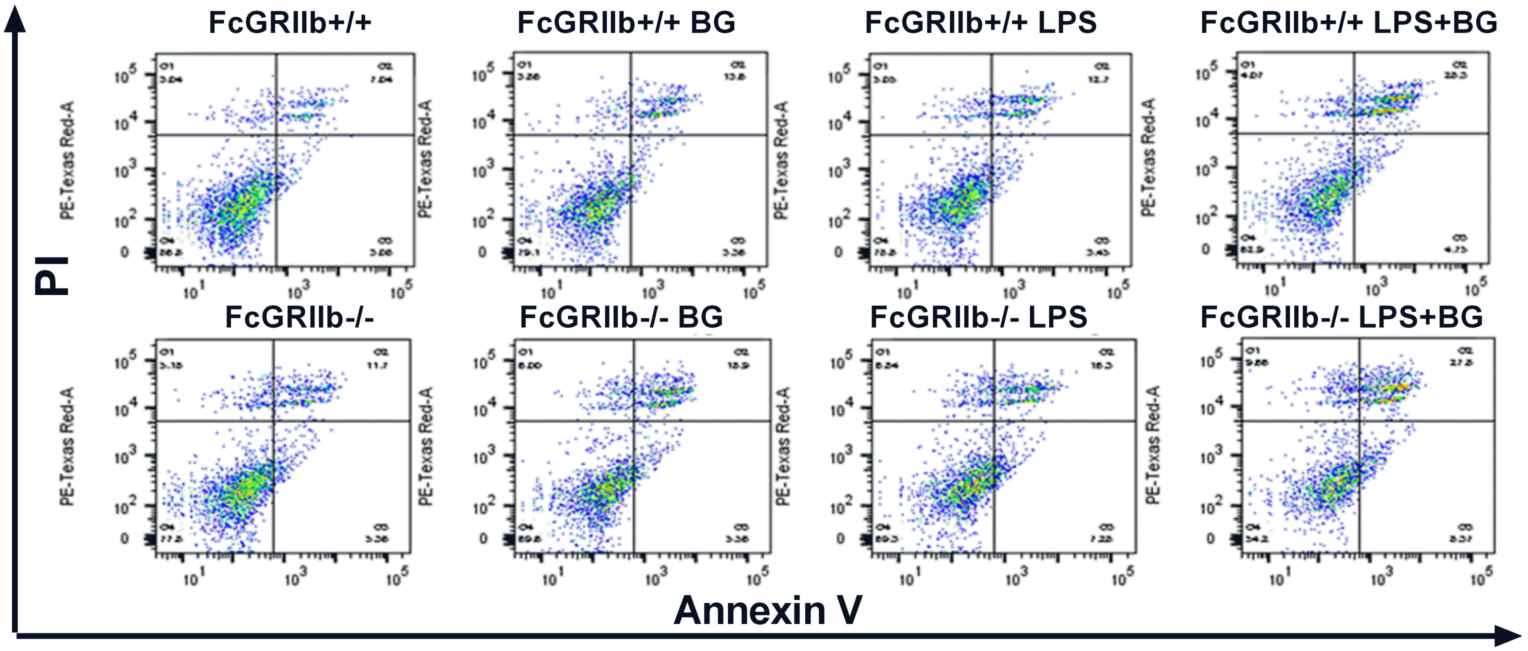

Supplementary Figure S3 Representatives of flow-cytometric analysis of FcGRIIb-/- macrophage after LPS-stimulation with and without BG following by cell starvation (see method) to determine necrotic cells (propidium iodide; PI +ve), early apoptosis cells (Annexin V +ve, PI –ve) and late apoptosis cells (Annexin V +ve, PI +ve).
